# Influence of stress in GaN crystals grown by HVPE on MOCVD-GaN/6H-SiC substrate

**DOI:** 10.1038/srep04179

**Published:** 2014-02-26

**Authors:** Lei Zhang, Jiaoxian Yu, Xiaopeng Hao, Yongzhong Wu, Yuanbin Dai, Yongliang Shao, Haodong Zhang, Yuan Tian

**Affiliations:** 1 State Key Lab of Crystal Materials, Shandong University, Jinan, 250100, P.R. China; 2 Department of Materials Science and Engineering, Qilu University of Technology, Jinan 250353, P. R. China

## Abstract

GaN crystals without cracks were successfully grown on a MOCVD-GaN/6H-SiC (MGS) substrate with a low V/III ratio of 20 at initial growth. With a high V/III ratio of 80 at initial growth, opaque GaN polycrystals were obtained. The structural analysis and optical characterization reveal that stress has a great influence on the growth of the epitaxial films. An atomic level model is used to explain these phenomena during crystal growth. It is found that atomic mobility is retarded by compressive stress and enhanced by tensile stress.

GaN has long been considered a most promising material for applications in short wavelength optoelectronics and high-power high-frequency electronics due to its excellent properties, such as a wide direct bandgap,high thermal stability and high electron velocities^1^,^2^,^3^. Currently, most GaN-based devices like light emitting diodes (LEDs) and laser diodes (LDs) have been commercialized by using hetero-epitaxial growth on sapphire, due to the lack of more compatible cost effective substrates. However, the large lattice mismatch (13.8%) and the large thermal expansion coefficient difference between GaN and sapphire results in a high dislocation density and residual stress, both of which seriously affect the optical and electrical performance of the fabricated devices^4^. A SiC substrate offers the advantages of small lattice mismatch, similar thermal expansion coefficent to GaN, and higher thermal conductivity. Thus, epitaxial growth of GaN on a SiC substrates is considered to be a very promising approach to solving these problems^5^,^6^,^7^,^8^. The thermal expansion coefficients, lattice constants, and thermal conductivity for GaN, 6H-SiC and sapphire are listed in Table 1^7^,^8^,^9^.

Lin et al. first reported the growth of 2.5–3.5 μm GaN films on a SiC substrate by molecular beam epitaxy (MBE) with a high electron mobility of 580 cm^2^/Vs^10^. The microstructure and defects in GaN films grown on a SiC substrate were also investigated^11^,^12^. Nikolaev et al. reported on GaN/6H-SiC p-n heterojunctions fabricated by the HVPE of GaN on p-doped 6H-SiC epitaxial layers grown by low temperature liquid phase epitaxy^13^. Insulating GaN layers doped with Zn were grown by HVPE on SiC substrates and the temperature dependence of the specific resistivity of the GaN:Zn layers was measured over the temperature range from 200 to 500 K^14^. H. Lahrèche et al. presented a three-step growth process that enabled them to grow high quality mirror-like GaN layers without using AlN buffer layers by MOCVD^15^. GaN/4H-SiC heterodiodes were fabricated where the GaN is directly grown by HVPE on off-axis 4H-SiC^16^. Lee et al. found that H-etching of the SiC substrate was important to eliminate stacking disorder in the GaN grown by MBE, and a high growth temperature reduced the density of screw dislocations in particular^17^. K. Jeganathan et al. demonstrated the growth of unstrained GaN layers on SiC-6H (0001) substrate by MBE using a double-step AlN buffer process grown at two different high temperatures with a difference of 30–50°C^18^. Our research group reported that GaN films were grown by HVPE on MOCVD-GaN/Al_2_O_3_ (MGA) and MOCVD-GaN/6H-SiC (MGS) samples. The strain variations were microscopically identified using Z scan Raman spectroscopy. The Raman peak (E_2_) shift indicates that the stress increased gradually as a function of increase in measurement depth^19^. The reported references mainly focused on the microstructures or defects of GaN films on a SiC substrate and the characteristics of GaN/6H-SiC p-n heterojunctions or heterodiodes grown by MBE or MOCVD. However, the influences of growth conditions and stress on GaN crystal growth with a MGS substrate grown by HVPE have not been reported. In this work, different growth conditions were tried for the growth of GaN on MGS substrates. The aim is to study the influence of stress in GaN crystals growth. We believe that this work may be helpful in understanding GaN crystal growth by HVPE on an MGS substrate.

## Experimental

GaN films were grown in a home-made vertical HVPE reactor. A template with a 5 μm GaN layer grown by MOCVD on the 6H-SiC substrates was employed as the starting substrate. Ga and NH_3_ were used as respective gallium and nitrogen sources. HCl gas was reacted with liquid Ga at 820°C to form GaCl, which was transported to the growth zone of the reactor and reacted with NH_3_ at 1030°C to form GaN molecules. Nitrogen was used as the carrier gas. The reactor pressure was kept at around atmospheric pressure. In this study, two different growth conditions (A and B, shown in Table 2) were used for the growth of GaN on the MGS substrate.

Two sets of samples were grown under identical conditions on MGA and MGS templates. The growth temperature was 1030°C. The GaN layers on the MGA and MGS templates have the same dislocation density (6 × 10^8^ cm^−1^). The gas flow rates of NH_3_ (800 mL/min, 1000 mL/min), HCl (10 mL/min, 20 mL/min), NH_3_ carrier gas (1000 mL/min), HCl carrier gas (1000 mL/min) and N_2_ (2000 mL/min) for growth condition A on MGA and MGS templates were the same. The gas flow rates of NH_3_ (400 mL/min, 1000 mL/min), HCl (20 mL/min, 20 mL/min, NH_3_ carrier gas (1000 mL/min), HCl carrier gas (1000 mL/min) and N_2_ (2000 mL/min) for growth condition B on MGA and MGS templates were the same. The heating rate and cooling rates were also the same. The surface morphology and structural quality of the as-grown GaN films were investigated by a variety of characterization techniques. AFM (Digital Instrument Dimension 3100) and FE-SEM (Hitachi S-4800) were used to investigate the surface morphology. Raman spectra of the samples were obtained by the LabRAM HR system of Horiba Jobin Yvon at room temperature using a 532 nm solid state laser as the excitation source. Photoluminescence (PL) measurements were carried out at room temperature using a 325 nm He–Cd laser as the excitation source. All samples were evaluated by high-resolution X-ray diffraction (HRXRD). The lattice parameters were determined by measuring the (002), (004) and (102), (204) peaks in a multi-diffraction ω-2θ scan.

## Results and Discussion

**Figure 1:**
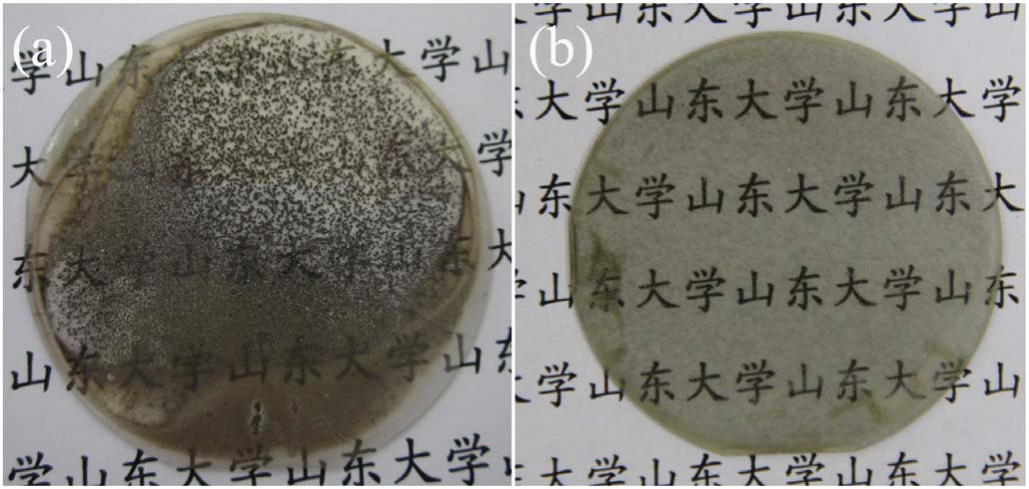
Photographs of the GaN films grown on MGS substrate under (a) condition A and (b) condition B.

Fig. 1 shows photographs of the GaN films grown on the MGS substrate under (a) condition A (high V/III ratio 80 at initial growth) and (b) condition B (low V/III ratio 20 at initial growth). Under growth condition A, (Fig. 1a) the surface is black and many small polycrystalline grains are observed. As shown in Fig. 1b, a 40 μm thick GaN layer with a mirror-like smooth surface was obtained on the MGS substrate under growth condition B.

**Figure 2:**
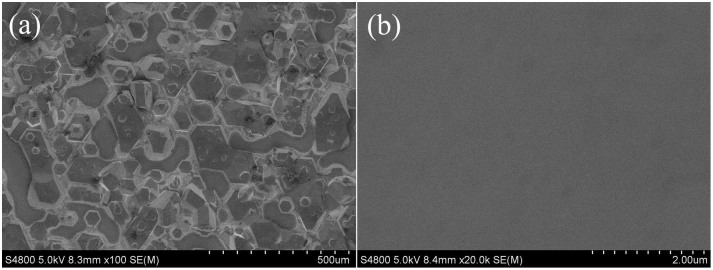
SEM images of GaN film surface morphology grown on MGS substrate under growth condition A (a) and B (b).

Fig. 2 shows SEM images of the surface morphology of the GaN films grown on MGS substrates under growth condition A (Fig. 2a) and B (Fig. 2b). In the image, many prismatic hexagonal grains are observed in the GaN under growth condition A, whereas for the GaN under growth condition B, a smooth pitless surface is observed. A GaN single crystal without cracks was successfully obtained under growth condition B. Using growth condition A, opaque GaN polycrystals were obtained. From the above results, we conclude that growth conditions have great influence on the MGS substrate.

**Figure 3:**
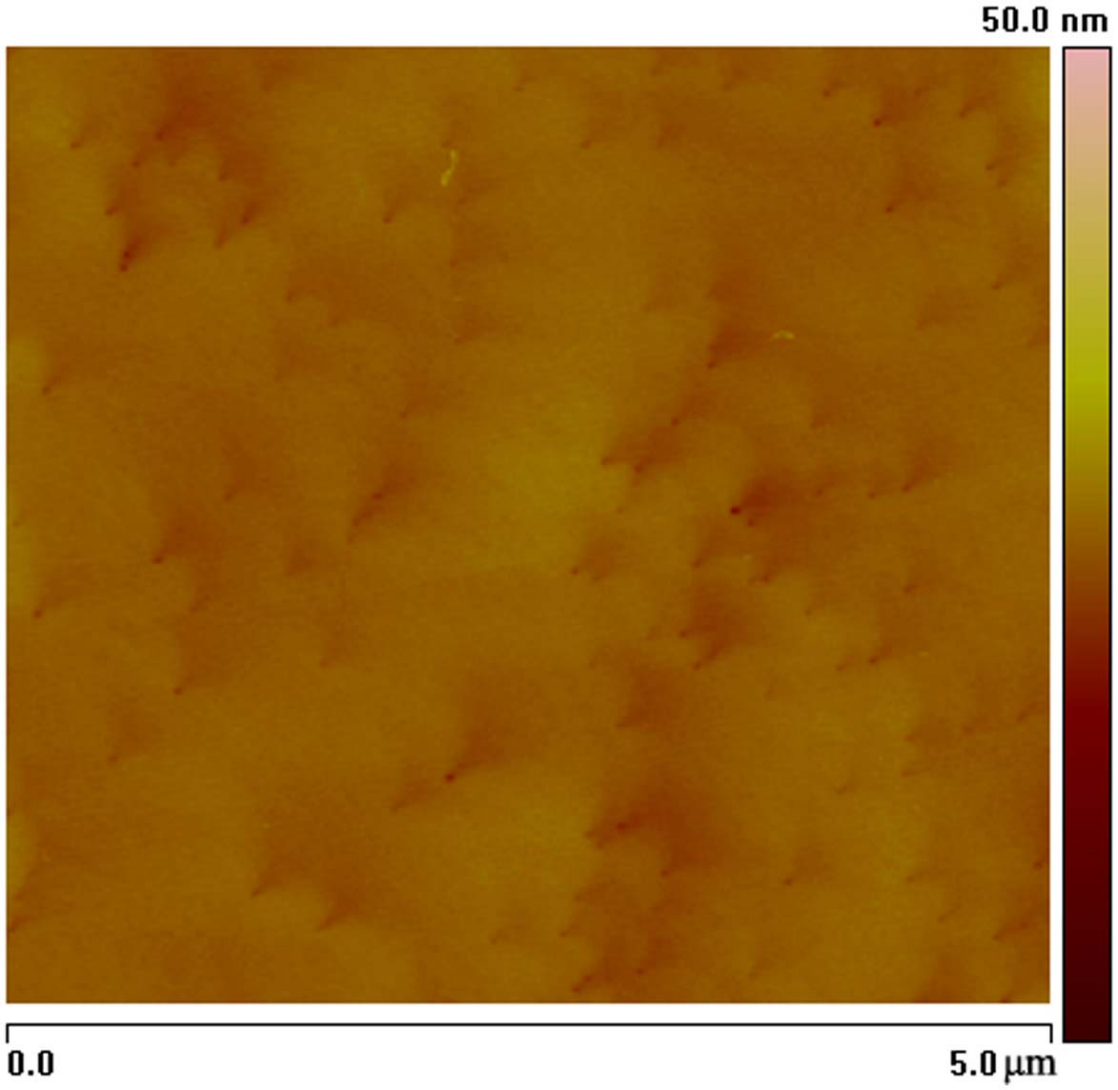
AFM image of surface morphology of GaN film grown on MGS substrates obtained with growth condition B.

Fig. 3 shows an AFM image of the GaN (growth condition A) epilayer in an area over a 5 μm × 5 μm area. A stepped terrace structure is observed and the RMS roughness is found to be 0.860 nm.

**Figure 4:**
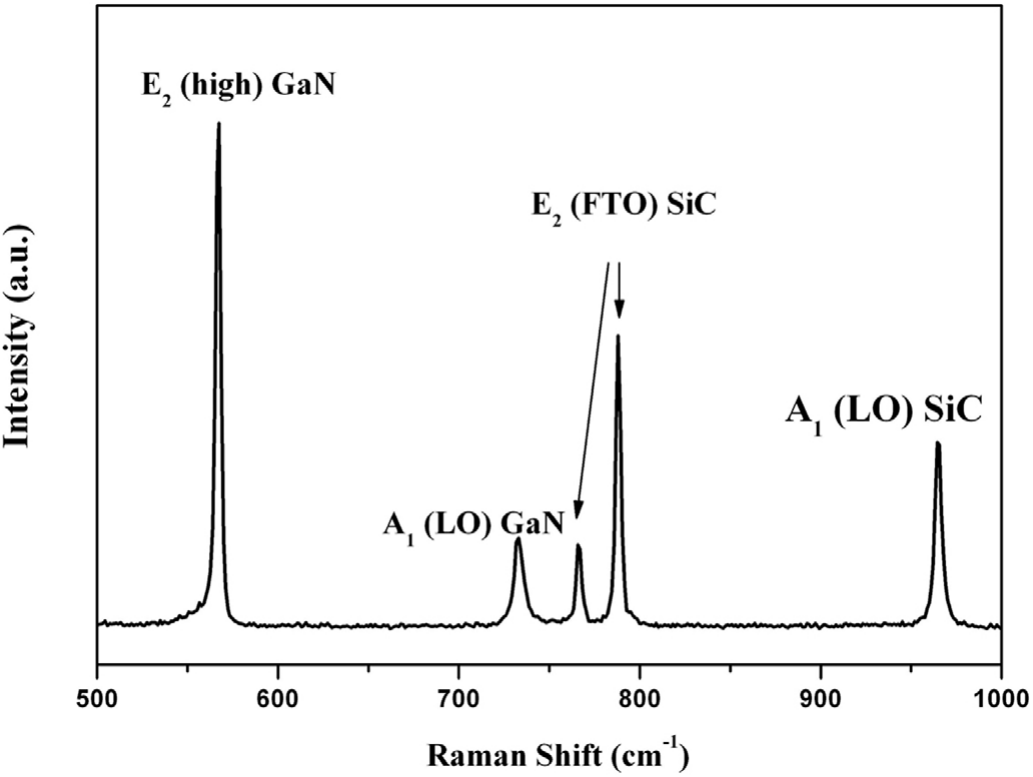
Raman spectrum of GaN film grown on MGS substrates under growth condition B.

Fig. 4 shows the Raman spectrum of a GaN films grown on the MGS substrate under growth condition B. The measurement was carried out with the z (xx) 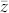 back scattering geometry. Five peaks are observed. In addition to the A_1_(LO) peak at 734.1 cm^−1^ and the E_2_ (high) peak at 568.7 cm^−1^ of GaN, the E_2_ (FTO) peaks of SiC are observed at 765.8 cm^−1^, 788.0 cm^−1^, and the A_1_ (LO) peak of SiC is observed at 964.5 cm^−1^. The E_2_ (high) phonon peak can be used to characterize the in-plane strain state of the GaN epilayer. The stress is calculated by the following equation^20^:
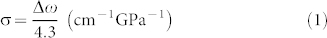
where *σ* is the biaxial stress and *Δω* is the E_2_ phonon peak shift. The E_2_ (high) phonon peak of stress-free GaN is believed to be located at 567.1 ± 0.1 cm^−1^ 21. The E_2_ (high) phonon peak of GaN grown on the MGS substrate is at 566.92 cm^−1^, indicating that a tensile stress of about 0.042 GPa was present.

**Figure 5:**
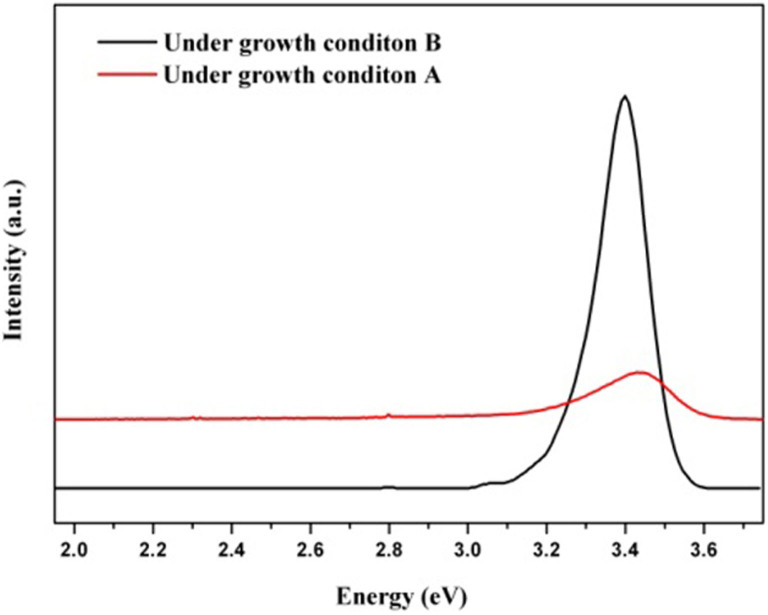
PL spectra of the GaN film grown on MGS substrates under growth conditions A and B.

The optical characteristics of the samples were obtained using PL measurements at room temperature (Fig. 5). The strong band-edge emission peaks of the GaN films grown on the MGS substrate can be observed near 3.399 eV. No yellow luminescence around 2.21 eV was observed. The luminescence peak of stress-free GaN is located at 3.471 eV^22^,^23^. The PL peaks of the samples exhibit a 72 meV red-shift with respect to those of completely relaxed bulk GaN, and indicate that the stress in GaN grown on the MGS substrate is tensile in nature. The narrow peak for the GaN film grown on the MGS substrate indicates that the crystalline quality of this layer is good. In contrast to the intensity obtained from the sample grown under growth condition B, the PL intensity from the polycrystalline GaN sample under growth condition A is very low. This observation implies that the concentration of non-radiative recombination centers is high.

**Figure 6:**
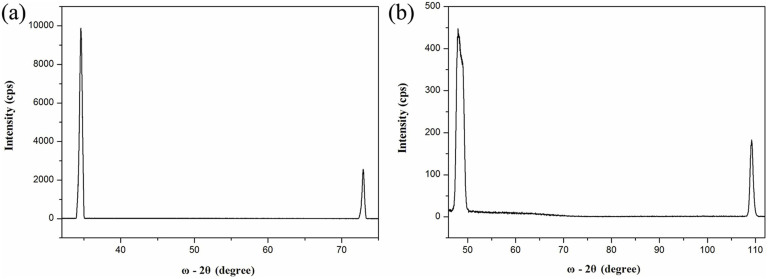
Diffraction peaks (ω-2θ scan) for planes (a) (002), (004) and (b) (102), (104) (b) of GaN film grown on MGS substrate under growth condition B.

Fig. 6 shows the XRD ω-2θ scan of the GaN film grown on the MGS substrate. The GaN (002), (004) and (102), (204) diffraction peak positions were used to calculate the lattice constants *c* and *a* by using the following equations^24^,^25^:
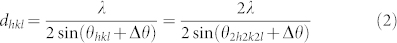

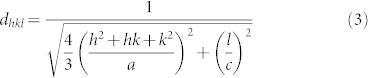
where (*h k l*) are the indices of the diffraction plane, *θ_h k l_* is the measured angular position of the (*h k l*) reflection, *Δθ* is the zero error of the instrument, and *λ* is the X-ray wavelength (0.154 nm for Cu *K_α1_* radiation). The in-plane strain was obtained by using the formula (4):
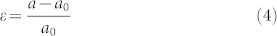
The strain-to-stress conversion is performed using the expression:
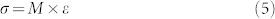
where *σ* is the in-plane stress, M (M_SiC_ = 602 GPa^26^, M_GaN_ = 478 GPa^26^) is the biaxial elastic modulus, and *ε* is the in-plane strain. The lattice constants of strain-free GaN are *a_0_* = 0.31892 nm and *c_0_* = 0.51850 nm^27^. The calculated lattice constants, strains and stresses are listed in Table 3. It can be seen that the GaN film on the MGS substrate has a smaller *c* value and larger *a* value compared to the strain-free parameters given the reference^27^. According to the calculated results, it can be concluded that the residual stress in GaN films grown on the MGS substrate is tensile in nature. The results are in agreement with the Raman and PL results.

**Figure 7:**
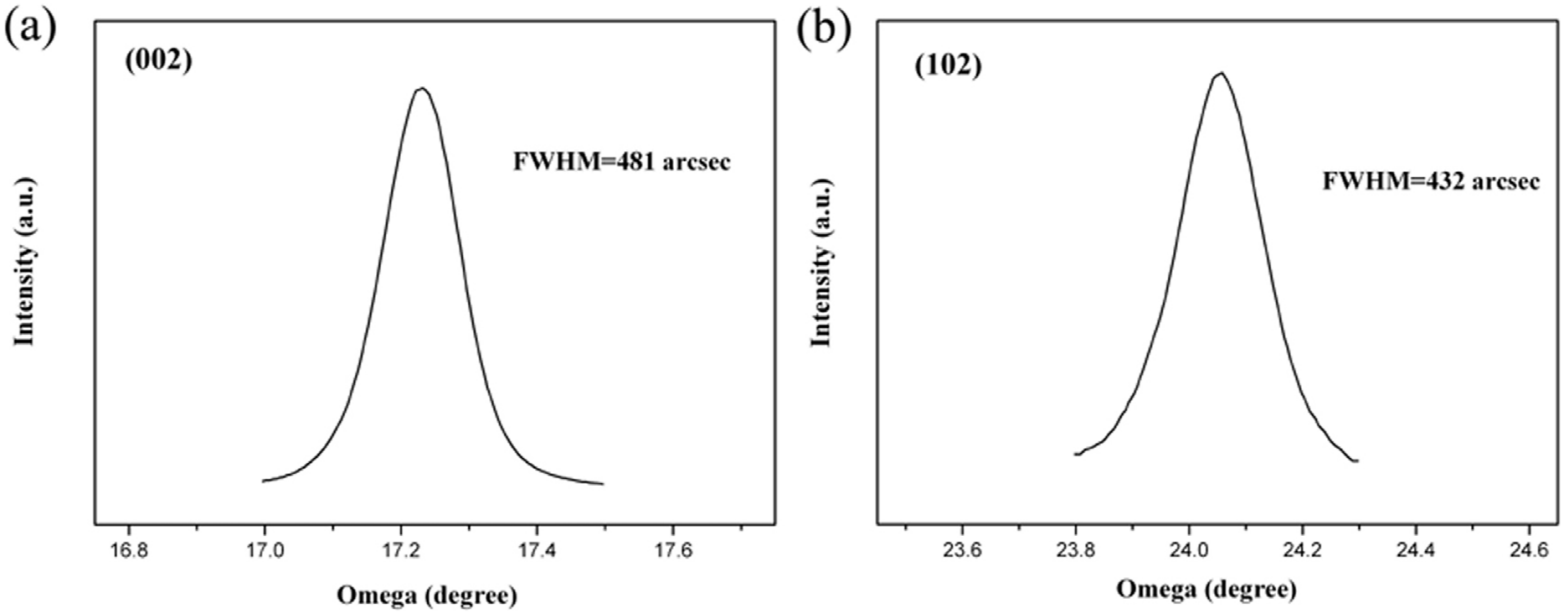
HRXRD rocking curves of GaN film grown on MGS substrate under growth condition B: (a) (002) ω-scans and (b) (102) ω-scans.

In addition, an HRXRD rocking curve for the (002) symmetric and (102) asymmetric planes of the GaN epilayer was performed to determine the crystal quality. The FWHM values of the symmetric (002) and asymmetric (102) diffraction line are 481 and 432 arcsec, respectively (Fig. 7).

Nucleation during HPVE is a complex process, which is usually dependent on temperature, substrate surface, gas flow rate, etc. The lattice perfection improved as the growth temperature increased, and the lattice quality and stoichiometry both reached optimum conditions as the N:Ga ratio approached a fixed value at a given growth temperature^28^. The diffusion barriers for a-plane and m-plane surfaces are different: on the a-plane surface, Ga ad-atoms exhibit substantially a smaller diffusion barriers along the *c*-axis, while on the m-plane surface, the Ga ad-atoms exhibit a substantially larger diffusion barriers along the *c*-axis^29^. In order to study the influence of stress in GaN crystals grown by HVPE on MGS substrates, the GaN growth process was in all cases carried out at the same growth temperature, Ga-pane growth surface, and gas flow rate. Both growth conditions A and B were used to grow GaN on a MGA substrate, and mirror-like smooth GaN films were obtained^19^,^30^. The stress condition in GaN grown on a MGA substrate is compressive in nature^19^, while on a MGS substrate it is tensile in nature. Therefore, we think that the different growth results obtained for the MGS substrate under conditions A and B are relevant to the stress present in the films.

**Figure 8:**
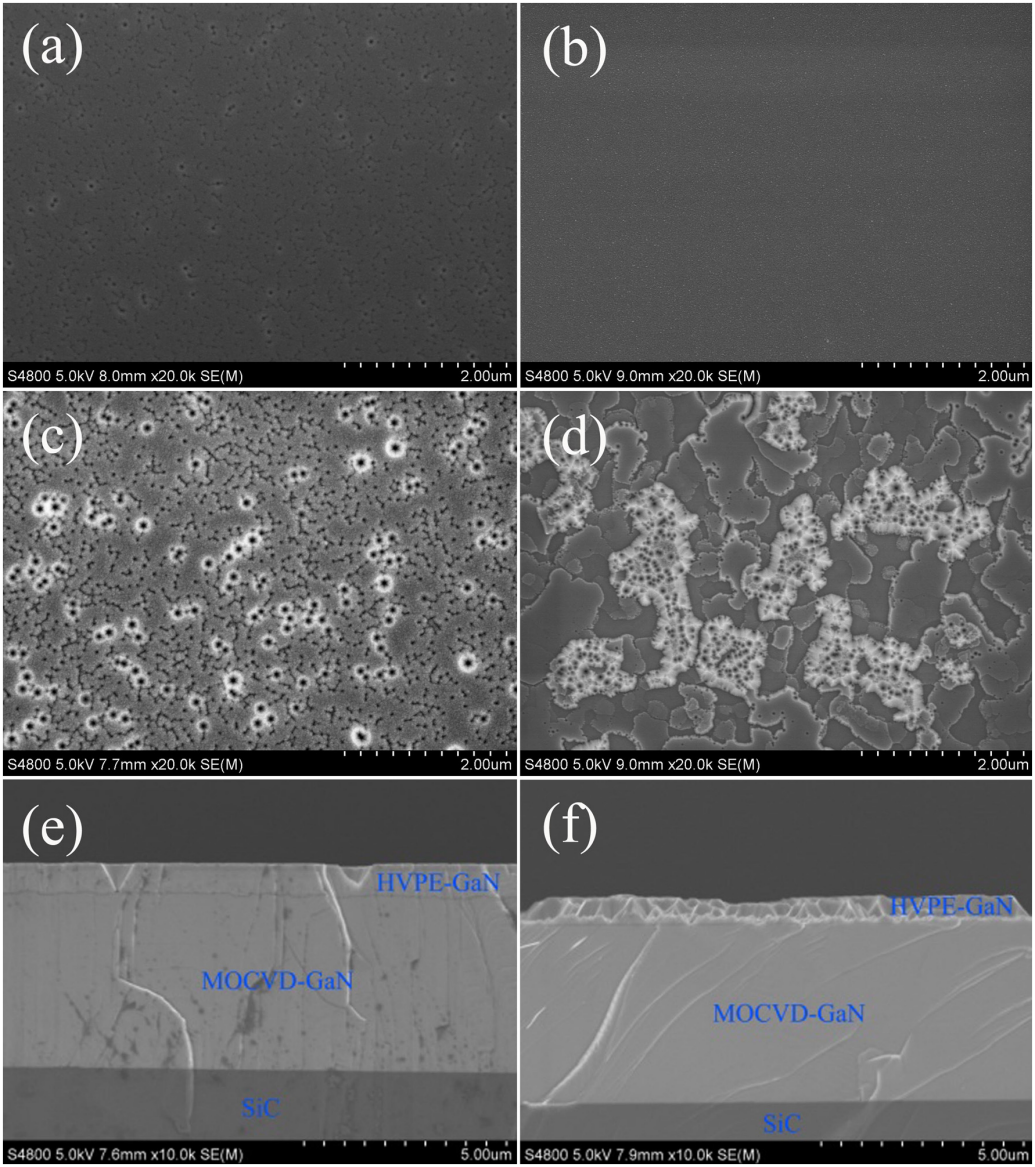
SEM images of GaN films grown on the MGA substrate under growth condition A (a) and B (b) with growth time of 20 min. SEM images of GaN films grown on the MGS substrate under growth condition A (c) and B (d) with growth time of 20 min. The cross-sectional SEM images of GaN films grown on the MGS substrate under growth condition A (e) and B (f) with growth time of 20 min.

The critical steps (V/III ratio = 80 for A and V/III = 20 ratio for B) in GaN growth for 20 min at 1030°C have been characterized by SEM (Fig. 8). Fig. 8a and Fig. 8b show SEM images of the GaN films grown on the MGA substrate. A continuous GaN layer was formed under growth conditions A and B. A lower V/III ratio enhances the three dimensional (3D) growth mode and a higher V/III ratio improves two dimensional (2D) growth mode^31^,^32^. For the MGA substrate, both 3D GaN nucleation layers with a low V/III ratio and 2D GaN nucleation layers with a high V/III ratio form coherent GaN films at the initial growth stage under compressive stress.

Fig. 8c and Fig. 8d show SEM images of GaN films grown on the MGS substrate. It can be seen that the GaN nucleation layers have been coalesced and continuous films are formed under growth condition A (Fig. 8c). Only a few pits were observed. However, many GaN islands were observed and the islands cannot coalescence with each other under growth condition B (Fig. 8d). Due to the tensile stress on GaN grown on the MGS substrate, the 3D GaN nucleation layers with low V/III ratio coalesce in the first growth step and smooth GaN films are formed with a high V/III ratio in the second step. However, the coalescence of 2D GaN nucleation layers with a high V/III ratio in the first growth step does not occur and prismatic hexagonal polycrystalline grains are formed instead. Fig. 8e and Fig. 8f show cross-sectional SEM images of GaN films grown on the MGS substrate. These results also confirmed that the growth mode is 2D under growth condition A and 3D under growth condition B for GaN grown on the MGS substrate.

Ad-atom surface mobility is considered to be a key parameter controlling the surface morphology^33^. It has been reported that stress in the film during growth can alter the surface mobility of ad-atoms and consequently the growth mode, thereby vastly influencing the surface morphology^34^,^35^,^36^,^37^. In the early stages, the GaN coalesces into islands. Then the islands are incorporated together by the Ga ad-atoms. The average diffusion length of the Ga ad-atoms plays an important role in the coalescence of the islands. The 3D growth mode usually produces many isolated islands because of the short diffusion length of the Ga ad-atoms^38^. The Ga ad-atoms only diffuse on the surface of the island, which allows the island structure to dominate. High dislocation density and rough surface morphology result. On the contrary, the 2D growth mode usually produces a smooth surface because of the long diffusion length of the Ga ad-atoms. The ad-atoms diffuse until they are incorporated into the island edges and allow the island to grow in the horizontal direction to form a smooth surface. The dislocations were bent, which dramatically reduced the threading dislocation density^38^.

The diffusion of an ad-atom on a flat surface is by far the most important kinetic process in film growth^39^. The diffusion coefficient is the measure of the ad-atom mobility. Ad-atom surface diffusion processes exhibit a characteristic diffusion length *L*, which is connected to the average atomic lifetime on the surface τ and the diffusion coefficient *D* (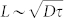). The process follows the Arrhenius equation: 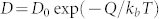, where *k_B_* is the Boltzmann constant, *T* is the temperature, and *Q* is the surface diffusion activation energy associated with the diffusion barrier^40^,^41^. The investigation of Jang et al. showed that the vacancy migration energy barrier (*E_m_*) is independent of pressure and remains almost constant^42^. So the activation energy barrier (*Q*), as the sum of vacancy formation enthalpy (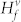) and the migration energy barrier (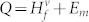), increases with increasing pressure^43^. The *Q* value with compressive stress is greater than that of the stress-free state. The *Q* value with tensile stress is less than that of the stress-free state. Therefore, it can be concluded that atomic mobility is influenced differently depending on the type of stress. The atomic mobility is retarded by compressive stress, but it is enhanced by tensile stress. A lower V/III ratio enhanced the 3D growth mode by increasing atomtic mobility, and a higher V/III ratio improved the 2D growth mode due to increased ad-atom mobility^31^. The stress condition in GaN grown on a 6H-SiC substrate is tensile in nature. For the MGS substrate, condition B (an increasing V/III ratio) was introduced in order to stabilize the atomic mobility and a smooth surface was obtained. Condition A (a decreasing V/III ratio) can caused disorder in the atomic mobility and opaque GaN polycrystals were obtained.

## Conclusions

The effect of stress on the growth of GaN films has been investigated. The atomic mobility is influenced differently depending on the type of stress, and consequently the growth mode is changed during GaN growth. It is found that 3D GaN nucleation layers grown at a low V/III ratio coalesced in the first growth phase and a GaN crystal was obtained on the MGS substrate. With a high V/III ratio in the initial growth step, opaque GaN polycrystals were obtained. Our work is helpful for understanding the growth of GaN crystals by HVPE on 6H-SiC substrate. GaN polycrystals can be avoided on the MGS substrate by using suitable growth conditions.

## Author Contributions

X.H. and Y.W. designed experiment. L.Z. wrote the main manuscript text. J.Y. carried out SEM measurements. Y.S., H.Z., Y.D. and Y.T. grew the sample. All authors reviewed the manuscript.

## Figures and Tables

**Table 1:** The coefficients of thermal expansion (*α_c_, α_a_*), lattice constants, and thermal conductivity (*κ*) for GaN, 6H-SiC and sapphire^7^,^8^,^9^

	*c* (nm)	*a* (nm)	*α_c_* × 10^6^	*α_a_* × 10^6^	*κ* (w/cmK)
GaN	0.5186	0.3188	3.17	5.59	2.1
6H-SiC	1.5117	0.3081	4.68	4.2	4.2
Sapphire	1.2991	0.4758	8.5	7.5	0.3

**Table 2:** Two different growth conditions (A and B) of GaN on MGS substrate

	Growth condition A		Growth condition B	
Growth parameter	First step (20 min)	Second step (4 h)	First step (20 min)	Second step (4 h)
V/III	80	50	20	50

**Table 3:** Calculated lattice constants, strains and stresses

	Grown on MGS substrate	Free standing GaN^27^
c (nm)	0.51843	0.51850
a (nm)	0.31899	0.31892
ε	0. 022%	-
σ	0.132 GPa	-
